# Separating Visuospatial from Visuomotor Coordination in Skill Estimation in Learning Disabled Children: The Eta-Mu Model

**DOI:** 10.7759/cureus.1901

**Published:** 2017-12-01

**Authors:** Carlo Aleci, Marzia Piccoli, Valentina Melotti, Elena Melis, Lorenzo Canavese

**Affiliations:** 1 Dept of Ophthalmology, The Gradenigo Hospital; 2 Dept of Ophthalmology, University of Turin, V.juvarra 19, 10100 Turin, Italy

**Keywords:** learning disability, visuoperceptive, motor coordination, rehabilitation, customization

## Abstract

Purpose

A model aimed at detecting the proportion of visuoperceptive and visuomotor coordination impairment in children with ascertained or suspected learning disability is described. The final purpose is to provide customized rehabilitation programs.

Methods

In this pilot study, four children (8-9 years) were administered a set of standardized tests to evaluate their ability to perform visuoperceptive and visuomotor tasks. Depending on the individual outcomes, two indexes have been computed from the resulting z-scores: η (Eta) that quantifies the visuoperceptive impairment, and μ (Mu) that expresses the alteration in visuomotor coordination.

Results

A condition of abnormality was evident in each patient: Subjects 1 and 3 suffered mainly from a visuoperceptive alteration (η higher than expected), while Subject 4 had reduced visuomotor coordination (μ higher than expected). Subject 2 showed balanced visuoperceptive and visuomotor impairment. Based on the obtained η and μ values, each child underwent a customized rehabilitation treatment, then they were examined again. At re-test, η or μ turned balanced and z-scores improved in the four patients.

Conclusions

The Eta/Mu model is effective in detecting the type of damage by quantifying the share of visuoperceptive and visuomotor coordination involvement in dyslexic children, allowing a customized rehabilitative approach. Such an approach, focused on treating the function found to be defective, appears to be effective in rebalancing individual visuomotor and visuoperceptive skills; it should, therefore, be taken into consideration when updating the rehabilitation plans of learning disabled children.

## Introduction

The integration of visual perception and motor coordination is crucial in school-age children for their academic development [1]. As far as we know, exams commonly used, like the Developmental Test of Visual Motor Integration [2], or the Supplemental Test of Visual Perception [3], as well as some items of the Visual Perception Test, assess the visuoperceptive and visuomotor function separately; in other words, the contribution of the two domains in determining the poor performance of the subject is not quantified. 

At best, visuoperceptive (VP) and visuomotor coordination (VMC) skills are measured separately with two complementary exams, then the final judgment is drawn considering the two different results [3]. In effect, to date, there is no way to precisely assess the share of the two domains in causing overall impairment, and to what extent such impairment depends on their defective integration. This is, indeed, a material point, since in our opinion, rehabilitation programs should be adapted or customized based on the findings in this paper; therefore, objective and accurate measures of VP and VMC to determine the co-operation of the respective functions is desirable. This study aims at addressing this issue, turning the z-scores obtained from a set of exams commonly used within the clinical setting into a VP and VMC abnormality index.

## Materials and methods

The model

A diagnostic set made of five different exams commonly used in our country has been selected; these are the Batteria per la Valutazione Della Memoria Visiva e Spaziale, i.e., Battery of Tests for Assessing Visual and Spatial Memory (BVS-Corsi) [4], Concise Assessment Scale for Children’s Handwriting (BHK) [5], Little Bell Test Revised (TCM) [6], the Rey-Osterrieth Complex Figure Test (ROCF)  [7-8], and the Developmental Eye Movement Test (DEM) [9-10].

The BVS-Corsi aims to evaluate different aspects of the visual and spatial working memory. It is made of first-level (screening purpose) and second-level trials. In this study, three second-level tests have been employed. The tests (visual memory, simultaneous, and sequential spatial memory) are presented on a liquid-crystal display (LCD) screen.

The BHK-concise assessment scale for children’s handwriting evaluates the fluency and speed of cursive handwriting. Subjects are required to copy a text in cursive handwriting within five minutes.

The TCM (Little Bell Test Revised) requires selective and sustained attention. Subjects are presented with four sheets, each showing a number of little bells embedded among other figures. The child is asked to mark each little bell as soon as possible. Speed and accuracy are measured.

In the Rey-Osterrieth Complex Figure Test, the child is asked to examine a complex nonsense figure, copy it, then reproduce it from memory.

The Developmental Eye Movement Test is used to assess the saccadic pattern in a condition comparable to reading. It is made of three subtests presented on a white sheet: the first and second subtests comprise two columns each made of 20 digits; the third subtest consists of an array of digits displaced in a random order. The subject is asked to read the digits in vertical, then in horizontal order. The time taken to complete the first and second subtests, as well as the time taken and number of errors/omissions in the third subtest are measured. The ratio score between the horizontal time (adjusted for the number of errors) and the vertical time is computed as an indicator of the oculomotor ability of the child.

The outcome of the exams is expressed as a z-score. Some tests of the set are expected to exclusively monitor the VP domain, while others are assumed to involve both VP and VMC, but to a different extent. In these cases, the proportion of VP and VMC involvement has been judged in advance. The judgment depends on an arbitrary, albeit accurately, pondered evaluation. In Table 1, the tests and the supposed VP/VMC proportion of recruitment are reported.

**Table 1:** The assumed proportion of visuoperceptive (VP) and visuomotor coordination (VMC) involvement in each test of the diagnostic set MLVS: Memoria di Lavoro Visuo-Spaziale BHK: Concise Assessment Scale for Children’s Handrwriting TCM: Little Bell Test revised ROCF: Rey-Osterrieth Complex Figure Test DEM: Development Eye Movement Test

Test and tested function	VP proportion of recruitment	VMC proportion of recruitment
Corsi MLVS visual memory	100%	0%
Corsi MLVS simultaneous spatial memory	100%	0%
Corsi MLVS sequential spatial memory	100%	0%
BHK-quality	30%	70%
BHK-velocity	30%	70%
TCM-velocity	80%	20%
TCM-accuracy	80%	20%
ROCF-copy	70%	30%
ROCF-memory	70%	30%
DEM-ratio score	100%	0%
Average proportion of recruitment	76%	24%

When completing the session, a global index of VP and VMC impairment, defined as η and μ respectively, is computed. The value of the two parameters, η and μ, depends on the z-score obtained in each test and on the proportion of VP/VMC involvement according to the following equations:

η= -Σ_n1…ni_ (VP_n_ * z score_n_) /n                          (Eq.1a)

μ = -Σ_n1…ni_ (VMC_n_ * z score_n_) /n                     (Eq.1b)

**Figure 1:**
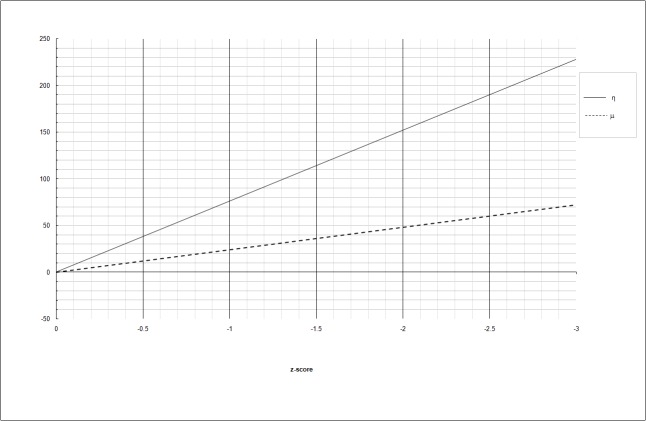
Increase of η (continuous line) and μ (dashed line) as a function of the average z-score Y-axis: η, μ arbitrary units

In case the performance of the child were normal (z-score = 0), η and μ are 0, suggesting that no impairment of the visuoperceptive and visuomotor coordination is present. As the performance of the child moves away from the normative value, η and  μ increase progressively. In the diagnostic set, the proportion of visuoperceptive recruitment is higher (76%) compared to the visuomotor coordination (24%); due to this difference, the slope of the regression model referred to η is steeper than the slope referred to μ (Figure 1).

The two linear trends refer to an ideal subject supposedly suffering from VP and VMC defects to the same extent. In this ideal case, the z-scores are identical for each exam, irrespective of the preponderant domain (VP or VMC) each test estimates; it follows that at a given level of z-performance, the resulting η and μ reflect the contribution required respectively to the VP and VMC components to normalize the z-score (i.e., to bring the average z-score to 0); as an example, for z-score of -1, the VP contribution is 76 and the VMC contribution is 24, reproducing the VP and VMC proportions captured by the diagnostic set. In this case, improving (i.e., lowering) the VP function by 76 η, or improving the VMC function by 24 μ, would normalize the average z-score. In case the average z-score was -2, the need for normalization would be double (152 η and 48 μ).

To summarize, the expected η and μ for a given average z-score in case the VP and VMC domain were equally affected, that is to say, if the z-score resulting from all the tests were the same, are computed as:

ηexp = 76 |z-score|                                 (Eq. 2a)

μexp = 24 |z-score|                                 (Eq. 2b)

Consider a child whose VP function is more affected than VMC. In this case, for z-score -1, the contribution required to the VP component to normalize the z-score (i.e., to make it 0) will be greater than in the previous hypothetical case (η will be higher than expected from the model). The opposite can be assumed for a child who suffers more from VMC than VP impairment: in this case, the contribution required to the VMC component to normalize the z-score (i.e., to make it zero) will be higher than in the previous, hypothetical case (μ will be higher than expected from the model).

As an example, let’s suppose the following outcomes (Table 2):

**Table 2:** Explanatory example for the model MLVS: Memoria di Lavoro Visuo-Spaziale BHK: Concise Assessment Scale for Children’s Handrwriting TCM: Little Bell Test revised ROCF: Rey-Osterrieth Complex Figure Test DEM: Development Eye Movement Test

Test and tested function (VP, VMC involvement)	z.score
Corsi MLVS visual memory (100,0)	-3
Corsi MLVS simultaneous spatial memory (100,0)	-2
Corsi MLVS sequential spatial memory (100,0)	-1.5
BHK- quality (30,70)	-0.5
BHK-velocity (30,70)	-0.5
TCM-velocity (80,20)	-1.5
TCM-accuracy (80,20)	-2
ROCF-copy (70,30)	-1.5
ROCF-memory (70,30)	-2
DEM-ratio score (100,0)	-2
Average z-score	-1.65
η_exp_	125.4
μ_exp_	39.6
Η	140.5
Μ	24.5

**Figure 2:**
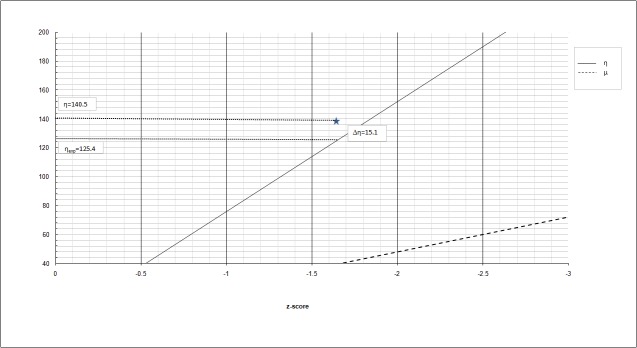
Prevalent visuoperceptive impairment Y-axis: η, μ arbitrary units

In this example, η_exp_ is 125.4 and μexp is 39.6 according to the equation (2a,b), while η is 140.5 and μ is 24.5 according to the equation (1a,b). Since η > η_exp_ and, in turn, μ<μexp, the global performance of the child reveals a prevalent visuoperceptive defect, with η above the expected value. The difference Δη between η and ηexp, therefore, quantifies the VP impairment. In this case, the VP deficit, expressed as Δη, is 15.1 (Figure 2).

Subjects

In this pilot study, four children aged eight and nine years (all males, third and fourth grade) suffering from specific learning disabilities were recruited. All subjects were normal at a preliminary orthoptic and ophthalmological examination. In all cases, visual acuity was 60/60 without correction. After completing the diagnostic set, average z-score and η and μ was computed in each subject.

All authors hereby declare that the experiment was performed in accordance with the ethical standards laid down in the 1964 declaration of Helsinki.

## Results

Results are summarized in the table below (Table 3):

**Table 3:** Results from the sample before rehabilitation. Subject 3 refused to undergo the Corsi test MLVS: Memoria di Lavoro Visuo-Spaziale BHK: Concise Assessment Scale for Children’s Handrwriting TCM: Little Bell Test revised ROCF: Rey-Osterrieth Complex Figure Test DEM: Development Eye Movement Test

Test and tested function (VP, VMC involvement)	Subj 1 z.score	Subj 2 z.score	Subj 3 z.score	Subj 4 z.score
Corsi MLVS visual (100,0)	-1.75	0.36	-	0.69
Corsi MLVS simultaneous (100,0)	-2.28	-0.37	-	1.21
Corsi MLVS sequential (100,0)	-0.19	1.48	-	-1.75
BHK- quality (30,70)	-0.11	0.3	0.21	-1.4
BHK-velocity (30,70)	-0.02	-0.6	-1.48	-1.22
TCM-velocity (80,20)	-2.4	-1.4	-0.44	-1.05
TCM-accuracy (80,20)	-3.5	-3.6	-3.28	-2.6
ROCF-copy (70,30)	-3.5	-1.2	-0.05	-0.65
ROCF-memory (70,30)	-3.8	-2	-2.31	-1.05
DEM-ratio (100,0)	0.06	-2.1	-0.78	-0.81
Average z-score	-1.75	-0.91	-1.16	-0.86
η_exp_	132.92	69.39	76.32	65.59
η	140.29	69.60	82.70	55.56
μ_exp_	41,98	21.91	39.82	20.71
m	34.61	21.70	33.44	30.74
Δ η	7.37	0.21	6.38	-10.03
Δ μ	-7.37	-0.21	-6.38	10.03

**Figure 3:**
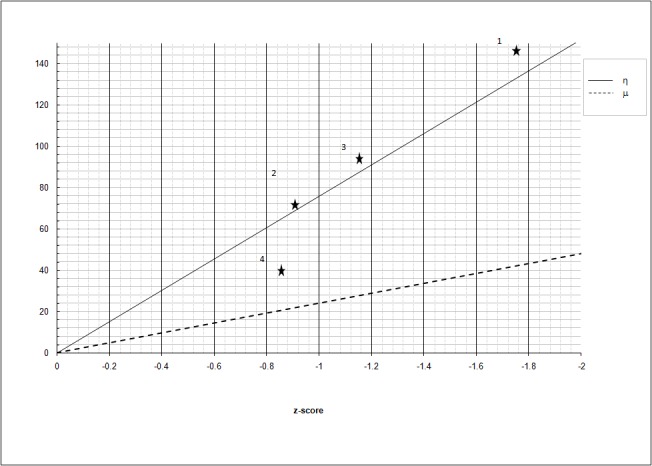
The values of η and μ relative to the 4 patients examined in the study Y-axis: η, μ arbitrary units

Patient 1 was the most affected by overall VP and VMC impairment, as suggested by his average Z-score. In this case, positive Δη (7.37) indicates VP defect, while negative Δμ suggests intact VMC. Patient 2 performed better, as shown by his average Z-score. In this case, Δη and Δμ are close to zero, indicating no defect in VP or VMC. His relatively poor performance in this case would therefore stem from abnormal VP/VMC integration. Patient 3 was moderately affected by overall VP impairment. Similar to the first case, positive Δη (6.38) indicates a VP defect. Finally, compared to the previous cases, Patient 4's positive Δμ of 10.03 suggests a defect in VMC (Figure 3).

Rehabilitation

Rehabilitation was carried out in the late morning, five days a week, for two weeks. Each rehabilitation session lasted 30 minutes.The rehabilitative program was customized based on the outcome of the prior evaluation, as follows:

*Patient 1*: Visual search of single items (digits, numbers, symbols, and words), copying/reproduction of  templates, search and comparison between letters and digits with different spatial orientation, visual and visuospatial memory training with images, visual attention training involving differences between images, training involving figure/ground segregation, rapid naming of letters and digits.

*Patient 2*: Reproduction of tridimensional models with monochromatic cubes, reproduction of tridimensional models with monochromatic cubes under different points of view, graphic reproduction of images, search and comparison between letters and digits with different spatial orientation, visual and visuospatial memory training with images, rapid naming of letters and digits.

*Patient 3*: Reproduction of tridimensional models with monochromatic cubes, graphic reproduction of images, copying/reproduction of templates, search and comparison between letters and digits with different spatial orientation, copy of complex geometrical images, visual attention training involving differences between images, rapid naming of letters and digits.

*Patient 4*: Visual search of single items (digits, numbers, symbols, words), copying/reproduction of templates, search and comparison between letters and digits with different spatial orientation, visual and visuospatial memory training with images, graphic reproduction of images, visual attention training involving differences between images, training involving figure/ground segregation, rapid naming of letters and digits, training focused on graphic fluency and precision, connecting dots to form an image.

The results after the rehabilitation are reported in Table 4.

**Table 4:** Results from the sample. Post-rehabilitation Due to incomplete pre-rehabilitation (Corsi test data from Patient 3; see Table 3), post-rehabilitation Patient 3 data has been excluded. MLVS: Memoria di Lavoro Visuo-Spaziale BHK: Concise Assessment Scale for Children’s Handrwriting TCM: Little Bell Test revised ROCF: Rey-Osterrieth Complex Figure Test DEM: Development Eye Movement Test

Test and tested function (VP,VMC involvement)	Subj 1 z.score	Subj 2 z.score	Subj 3 z.score	Subj 4 z.score
Corsi MLVS visual (100,0)	-0.71	0.69	-	0.18
Corsi MLVS simultaneous (100,0)	10.72	1.19	-	-1.43
Corsi MLVS sequential (100,0)	-1.18	0.05	-	-0.66
BHK-quality (30,70)	0.54	0.61	0.05	-1.59
BHK-velocity (30,70)	-1.16	-1.71	-0-48	-0.06
TCM-velocity (80,20)	-0.97	-0.7	1.8	0.5
TCM-accuracy (80,20)	-1.66	-0.27	-0.78	-0.15
ROCF-copy (70,30)	1.15	1.26	-1.58	-1.51
ROCF-memory (70,30)	-0.3	0.57	-1.29	-2.14
DEM-ratio (100,0)	0.47	-3.05	-1.15	-0.39
Average z-score	-0.21	-0.14	-0.49	-0.73
η_exp_	15.96	10.34	32.20	55.10
η	13.95	9.45	35.31	50.70
μ_exp_	5.04	3.26	16.80	17.40
m	7.05	4.15	13.69	21.08
Δ η	-2.01	-0.89	3.11	-4.40
Δ μ	2.01	0.89	-3.11	4.40

**Figure 4:**
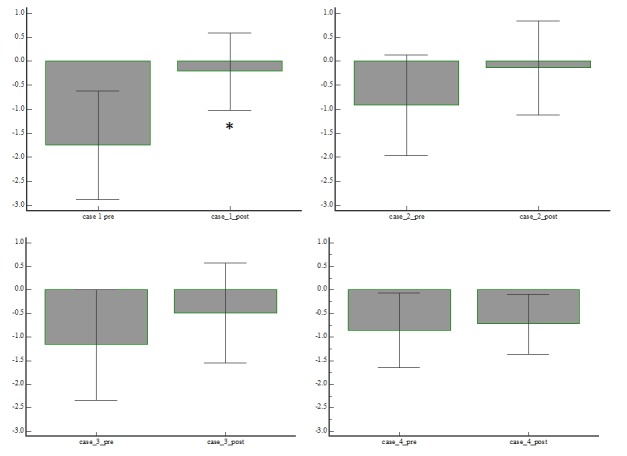
Average z-score before and after the rehabilitation program

At the end of the training program, the average z-score of the whole sample tended toward normalization (pre-rehabilitation: median z-score: -1.03, IR: from -1.45 to -0.88; post-rehabilitation: median z-score: -0.35, IR: from -0.61 to -0.17); even if the overall performance improved in all cases, such improvement was statistically significant only in Patient 1, that is, in the subject showing the worst performance (Figure 4).

**Figure 5:**
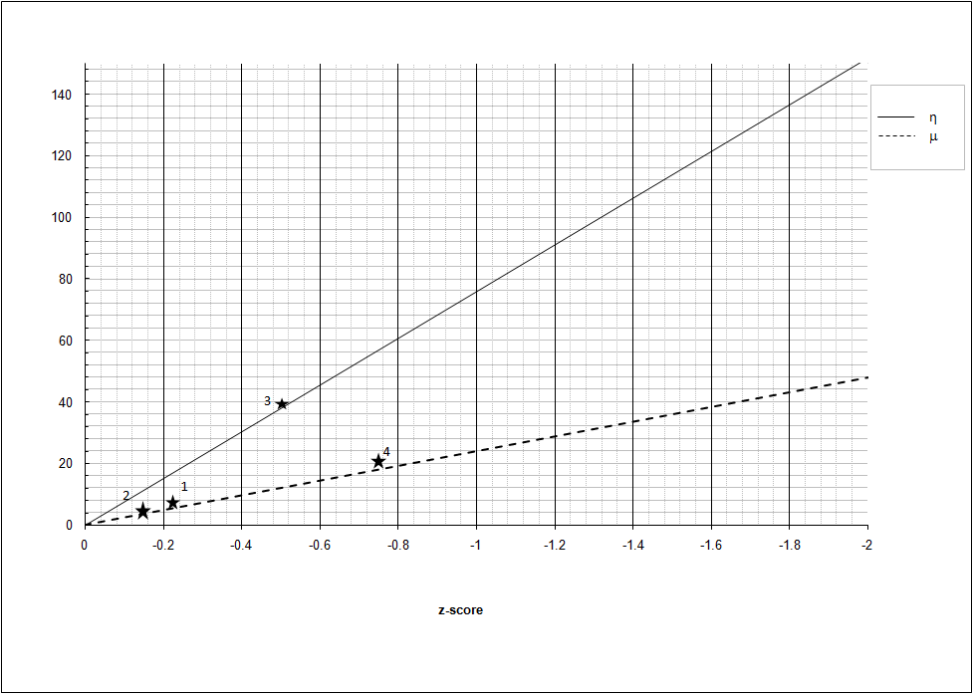
Change in visuoperceptive (η) and visuomotor coordination (μ) in the four patients after the rehabilitation. Compare with Figure 3. Y-axis: η, μ arbitrary units

The change in visuoperceptive and visuomotor coordination share after the rehabilitation is reported in Figure 5.

By inspecting the graph, it can be observed that in Subject 1 consistent improvement of VP performance has been obtained, causing normalization of the z-score.

In Subject 2, the customized practice described above improved overall performance in the administered tests, with VP and VMC skills remaining balanced. Such improvement would lead to normalization of the z-score.

In Subject 3, the practice improved VP performance, and such improvement can be correlated to a reduction of the z-score.

Finally, there was mild improvement of VMC function in Subject 4; however, it was not statistically significant to improve the z-score. 

## Discussion

The main requirement for an effective rehabilitation program for learning disabled children is assessing their altered visuoperceptive and visuomotor functions. Not only the impairment of one of the two domains, but also their defective integration may exacerbate the problem.

The model advanced in this preliminary paper aims to detect whether the z-deviation from the normality range in skills requiring the efficient cooperation of the visual and motor system relies on a preponderant visuoperceptive or visuomotor defect, or if it depends mainly on their reciprocal integration.

Of the children recruited in the experiment, Subjects 1 and 4 showed consistent VP/VMC imbalance, with prevalent involvement of the VP or VMC domain, respectively. It should be noted that in the small sample, Subject 1 is the more affected case, while Subject 4 is the case closest to normal, suggesting that the amount of selective VP/VMC deficit is independent of the overall degree of impairment (expressed as average z-score). This finding is supported by lack of correlation (r: -0.61, p: .10), and demonstrates that the outcome of the Eta Mu model is not biased by the global performance of the patient.

In turn, Subject 2 scored as predicted by the model; that is to say, he showed balanced performance in the VP and VMC domain. This finding suggests that the source of the alteration relies in the integration process.

In all children, the z-score improved after the rehabilitation program. In this respect, the failure to achieve statistical significance could be due to the small size of the sample.

The ameliorative effect seems to depend on the degree of impairment at baseline: as a matter of fact, it reached a significant value only in the subject with the worst performance (Subject 1).

The patient who showed prevalent VMC deficit (Subject 4) was the least responsive to the treatment. Non-optimal customization of the rehabilitation program might explain the failure. One theory for this might be that VMC impairment is more rehabilitation-resistant compared to VP impairment. In support of this hypothesis, the proportion of improvement in the VP domain after treatment ranged from 90% (Case 1) to 48.7%% (Case 3), while Subject 4 showed enhanced VMC function by only 8.7%. However, more observations are needed to support this theory currently.

## Conclusions

In conclusion, the Eta/Mu model may be utilized in the future to determine learning disabilities in children. As seen in this paper, it can do so by its predictive ability to investigate impairments and defective integration of visuoperceptive and visuomotor coordination. Further studies are required to better understand the potential of the model, thereby its usefulness in helping develop customized rehabilitation programs.
